# Effect of oral metformin on gut microbiota characteristics and metabolite fractions in normal-weight type 2 diabetic mellitus patients

**DOI:** 10.3389/fendo.2024.1397034

**Published:** 2024-08-27

**Authors:** Xiaohong Niu, Ying Wang, Linqing Huang, Pengna Guo, Shi Zhang, Yan Sun, Miaomiao Jin

**Affiliations:** ^1^ Endocrine and Metabolic Pathology, Shanxi Medical University, Taiyuan, China; ^2^ Department of Endocrinology, Changzhi Medical College Affiliated Heji Hospital, Changzhi, China; ^3^ Endocrine and Metabolic Pathology, Changzhi Medical College, Changzhi, China

**Keywords:** type 2 diabetes mellitus, metformin, metabolomics, gut microbiota, body mass index

## Abstract

**Background and aims:**

To analyze the effect of oral metformin on changes in gut microbiota characteristics and metabolite composition in normal weight type 2 diabetic patients.

**Methods:**

T2DM patients in the cross-sectional study were given metformin for 12 weeks. Patients with unmedicated T2DM were used as a control group to observe the metrics of T2DM patients treated with metformin regimen. 16S rDNA high-throughput gene sequencing of fecal gut microbiota of the study subjects was performed by llumina NovaSeq6000 platform. Targeted macro-metabolomics was performed on 14 cases of each of the gut microbiota metabolites of the study subjects using UPLC-MS/MS technology. Correlations between the characteristics of the gut microbiota and its metabolites, basic human parameters, glycolipid metabolism indicators, and inflammatory factors were analyzed using spearman analysis.

**Results:**

Glycolipid metabolism indexes and inflammatory factors were higher in normal-weight T2DM patients than in the healthy population (*P*<0.05), but body weight, BMI, waist circumference, and inflammatory factor concentrations were lower in normal-weight T2DM patients than in obese T2DM patients (*P*<0.05). Treatment with metformin in T2DM patients improved glycolipid metabolism, but the recovery of glycolipid metabolism was more pronounced in obese T2DM patients. None of the differences in α-diversity indexes were statistically significant (*P*>0.05), and the differences in β-diversity were statistically significant (*P <*0.05). Community diversity and species richness recovered after metformin intervention compared to before, and were closer to the healthy population. We found that *Anaerostipes*/Xylose/Ribulose/Xylulose may play an important role in the treatment of normal-weight T2DM with metformin by improving glycemic lipids and reducing inflammation. And Metformin may play a role in obese T2DM through *Romboutsia*, medium-chain fatty acids (octanoic acid, decanoic acid, and dodecanoic acid).

**Conclusion:**

Gut microbial dysbiosis and metabolic disorders were closely related to glucose-lipid metabolism and systemic inflammatory response in normal-weight T2DM patients. Metformin treatment improved glucose metabolism levels, systemic inflammation levels in T2DM patients, closer to the state of healthy population. This effect may be mediated by influencing the gut microbiota and microbial host co-metabolites, mainly associated with *Anaerostipes* and xylose/Ribulose/Xylulose. Metformin may exert its effects through different pathways in normal-weight versus obese T2DM patients.

## Introduction

Type 2 diabetes mellitus (T2DM) is a metabolic disease characterized by prolonged hyperglycemia due to disorders of glucose metabolism caused by insulin deficiency and/or impaired insulin bioactivity, often accompanied by a variety of metabolic disorders, which seriously jeopardizes the life and health of patients. Some studies (1) have shown that the increase in the number of type 2 diabetes patients worldwide is related to factors such as overweight and obesity, and its mechanism may be its own insulin resistance. However, there is growing evidence that T2DM can also occur in people with normal body mass index (BMI) that and that normal-weight patients with T2DM exhibit a higher propensity for pancreatic β-cell failure, as well as having lower waist circumference, waist-to-hip ratio, total and visceral fat levels, and fasting insulin, fasting C-peptide, and insulin resistance indices (HOMA2-IR) compared with obese T2DM (2, 3). Since normal-weight T2DM patients do not require weight-loss treatment and their metabolic disorders are less severe than those of obese T2DM patients, they require different treatments compared with obese T2DM patients. Currently, most of the studies at home and abroad have been conducted on obese or overweight T2DM patients, and there is a lack of literature support for studies on normal-weight T2DM patients; therefore, finding more effective hypoglycemic agents in glucose-lowering regimens for normal-weight T2DM patients has become a hot topic in current research.

It is well known that gut microbiota plays an important role in energy acquisition from food and immunomodulation. As an invisible organ in the human body, the dysregulation of gut microbiota is involved in the development of various metabolic diseases in the human body, such as obesity, insulin resistance, T2DM, inflammatory bowel disease, and non-alcoholic fatty liver disease, etc. The short-chain fatty acids (SCFA) and amino acid metabolism derivatives produced by gut microorganisms can also act as messengers to regulate the body’s glucose metabolism and affect the development of T2DM. Some studies have found that in healthy populations, the gut microbiota of normal-weight and obese populations had significant differences, with the abundance of the thick-walled phylum in normal-weight populations being lower than that in obese populations, and the abundance of the phylum Bacteroidetes being higher (4). The results showed that the composition of the gut microbiota and its metabolites were significantly different between diabetic obese mice and normal-weight mice (5). While the gut microbiota metabolites of short-chain fatty acids, fecal bile acid concentrations were significantly lower, and phosphatidylcholine (PC), lysophosphatidylcholine (LPC), and palmitocarnitine were elevated in patients with T2DM compared with healthy subjects (6). Therefore, we venture to predict that there may be a difference in gut microbiota between normal-weight and obese patients in the T2DM population, but there is a lack of literature support regarding normal-weight T2DM patients in population-based studies to be further explored.

Metformin is the most widely used hypoglycemic agent for the treatment of patients with T2DM, and is recommended as the first-line therapeutic agent because of its obvious hypoglycemic effect, relative safety, and low cost. The antidiabetic mechanism of action of metformin may be through the activation of adenosine monophosphate-activated protein kinase (AMPK), inhibition of hepatic glucose gluconeogenesis, increase insulin sensitivity, and enhancement of peripheral glucose uptake in the liver and skeletal muscle. Currently, it had been shown that the efficacy of metformin was not related to body weight, but Deng et al. found different results in their study, showing that BMI was negatively correlated with the magnitude of decrease in glycosylated hemoglobin and fasting glucose after metformin intervention (7, 8). Recent studies showed that metformin significantly improved the gut microbiota of T2DM patients, which may promote the improvement of immune status through the modulation of gut microbiota, which in turn exerts a glucose-lowering mechanism, but this interaction is still unclear (9). Thomas (10) and Ilze (11) et al. showed that the composition of the gut microbiota of healthy young men changed significantly with metformin, such as a decrease in the abundance of Enterobacteriaceae and an increase in the abundance of Prevotella and Bifidobacteria, but returned to baseline levels after discontinuation of the treatment, suggesting that metformin had a significant effect on the gut microbiota of healthy populations, and that the effect disappeared after discontinuation of the drug. Similarly, in a double-blind trial, we observed a decrease in α diversity and an increase in Ehrlichia, Bifidobacterium, and Akkermannia and a decrease in Enterobacteriaceae in obese T2DM patients treated with metformin (11). However, what is the effect of metformin on the gut microbiota of normal-weight T2DM patients, and whether it can improve metabolic indexes such as blood glucose in normal-weight T2DM patients by regulating the gut microbiota needs to be further explored. In addition, it had also been found that Metformin promoted changes in gut microbial metabolites in T2DM patients, and the results of macrogenomics and metabolomics analyses revealed that lipopolysaccharide biosynthesis was increased and significantly increased butyrate and propionate in the gut of T2DM patients (11). Therefore, we can propose a hypothesis that the application of metformin therapy in normal-weight T2DM patients may be through the pathway of gut microbiota and its metabolites, but not the same as in obese T2DM patients.

In summary, our study intends to observe the characteristics of gut microbiota in normal-weight T2DM patients, and to explore the effects of gut microbiota and its metabolites on the body’s glucose-lipid metabolism after metformin intervention, so as to explore the pathogenesis of the disease and the antidiabetic effect of metformin, and to explore the correlation between them. It will provide a basis for further revealing the relationship between diabetes and gut microbiota and guiding the precise treatment program for normal-weight T2DM patients.

## Materials and methods

### Participants

This study was a cross-sectional plus prospective study. Cross-sectional study:60 T2DM patients with a disease duration of ≤5 years admitted to the outpatient clinic or inpatient department of the Department of Endocrinology of Changzhi Medical College Affiliated Heji Hospital from December 2018 to June 2023 (30 cases in the obese group with a BMI of ≥28 kg/m^2^ and 30 cases in the normal group with a BMI of 18.5 kg/m^2^ ≤ 24 kg/m^2^) were selected as the study subjects, and a healthy population was selected from those who had a medical examination at the medical checkup center of the hospital during the same period as a normal control persons (NCP). Healthy people were selected as the normal control persons (NCP).Written informed consent was obtained from all participants prior to enrollment. The study was performed in adherence to the principles of the Declaration of Helsinki with regard to ethical research involving human subjects, and study protocols were approved by the Medical Ethics Committee of Heji Hospital (June 4, 2020, Approval no. 4).

NNT group (n=30): normal-weight T2DM patients;UNT group (n=30): obese T2DM patients;NCP group (n=20): normal control persons. Prospective study: Sixty patients with T2DM from the cross-sectional study were given metformin alone for 12 weeks. NMT group (n=30): metformin monotherapy in normal-weight T2DM patients; MET group (n=30): metformin monotherapy in obese T2DM patients. Medication regimen: Metformin 0.5g/dose 3 times/day with meals, or adjusted according to blood glucose, maximum dose not exceeding 2g/day.

Inclusion criteria for patients with T2DM: 1.Patients with T2DM meeting the diagnostic criteria for diabetes mellitus recommended by the WHO in 1999;2.11% > glycated hemoglobin (HbAlc) ≥6.5%;3. Meet the diagnostic criteria for obesity in China: BMI (kg/m²) ≥28 and waist circumference ≥85cm for men and ≥80cm for women, and meet the diagnostic criteria for normal body weight in China: 18.5kg/m^2^ ≤ BMI <24kg/m^2^ and waist circumference <85cm for men and <80cm for women;4. Age between 18-75 years old;5. Disease duration ≤5 years, no previous use of any hypoglycemic drugs;6. No application of antibiotics, microbial live bacterial preparations, etc. in the last 6 months. Inclusion criteria for the NCP population: 1. Normal results of the oral glucose tolerance test, normal people with no diabetes mellitus and no family history of autoimmunity;2. Meets the diagnostic criteria for normal body weight in China: 18.5kg/m^2^ ≤BMI <24kg/m^2^, and age, gender, and geographic area are matched with T2DM patients.

Exclusion criteria for T2DM patients:1. type 1 diabetes mellitus, diabetic acidosis, patients with diabetic gastroparesis, hidden autoimmune diabetes mellitus in adults, gestational diabetes mellitus and other types of diabetes mellitus etc.;2.Suffering from severe hepatobiliary, gastrointestinal diseases, such as, inflammatory bowel disease, ulcerative colitis, Crohn’s, bacillary dysentery, intestinal obstruction, etc., without history of pancreatitis, history of constipation, alcoholism;3. Long-term use of antibiotics and microbial preparations. Patients who used antibiotics (>7 days) or supplemented with live microbial agents (>7 days) within 6 months of sampling were excluded from the study. If the above agents were used for a short period of time (≤7 days), they could be withdrawn from the study for 4 weeks and then re-investigated.;4. Hypersensitivity to GLP-1RA and metformin and its adjuvants;5. Renal insufficiency, blood creatinine level: male >132.61umol/L (1.5mg/dl), female >123.8umol/L (1.4mg/dl), or glomerular filtration rate [GFR<60ml/(min×1.73m^2^)], serum ALT or AST exceeds the upper limit of normal more than 3 times, or have severe hepatic insufficiency;6. History of surgery and trauma within the last six months;7. Previous medullary thyroid carcinoma or family history of medullary thyroid carcinoma, multiple endocrine neoplasia syndrome, combined with severe endocrine system diseases such as thyroid, adrenal gland, pituitary gland, etc.; (e.g. medullary thyroid carcinoma or family history of medullary thyroid carcinoma, multiple endocrine neoplasia syndrome);8. Pregnant and lactating women;9. Have used or are using drugs that may affect body weight within 3 months;10. Combined malignant tumor, acute or chronic infectious stage of disease, infectious disease and other chronic wasting disease, mental and psychological disease, drug or other drug abuse; combined with severe cardiopulmonary and cerebral insufficiency, such as respiratory failure, heart failure, myocardial infarction, severe cerebrovascular disease, and so on.

### Data and sample collection

Socio-demographic information (name, sex, age, date of birth) was collected from all the study subjects, and detailed inquiries were made about current medical history, past history, family history, behavioral and lifestyle habits (diet, exercise, smoking, alcohol consumption, etc.). Height and weight were measured early in the morning on an empty stomach, after removing shoes and jacket, and BMI was calculated. The patient was instructed to stand in a standing position after urination, with feet 25-30 cm apart, hands naturally hanging down, maintaining normal breathing, and relaxing the trouser belt. The waist circumference was measured at the end of exhalation and inhalation with a soft tape measure around the abdomen through the midpoint of the line between the anterior superior iliac spine and the lower edge of the 12th rib. The examinee was instructed to empty the bladder, rest for 10 minutes to take a sitting position, arms naturally placed on the desktop, the upper arm should be placed at the same level as the heart, the right upper limb was measured 2 times consecutively and then the average value was taken.

Collection of biochemical indexes: Fasting and fasting blood was collected from the subjects and tested in the Laboratory Department of Heji Hospital affiliated to Changzhi Medical College, the indexes included: glucose metabolism indexes: fasting glucose (FBG), fasting C-peptide (FCP), glycosylated hemoglobin (HbAlc), lipid metabolism indexes: total cholesterol (TC), total triglyceride (TG), low-density lipoprotein cholesterol (LDL-C), high-density lipoprotein cholesterol (HDL-C).

Dietary education: all T2DM patients were provided with diabetes health education and guidance before and after enrollment and during the experiment. Specifically: according to the dietary recommendation standard of medical nutrition therapy, standard weight was calculated according to the formula of adult standard weight: adult standard weight = height (cm) - 105, according to the type of patient’s physical activity (bed rest, light physical labor, moderate physical labor, heavy physical labor), combined with the calorie supply for adult diabetic patients recommended by the medical nutrition therapy (kcal/kg of standard body weight), standard calories were calculated, and the proportion of nutrients (carbohydrate calories accounted for 55-65% of total calories, fat calories accounted for 55-65% of total calories) was calculated. Standard calories were calculated, and the proportion of nutrients (55-65% of total calories from carbohydrates, 20-25% of total calories from fat, and 10-20% of total calories from proteins) was carried out to calculate the proportion of each nutrient.

Collection of fecal specimens: fecal samples were collected on the morning of the day they were received, and study participants collected fecal samples in sterile and sealed fecal collection tubes provided by the study team and sealed with the preservation solution that came with the collection tubes. They were also immediately stored in the laboratory in a -80°C refrigerator until analysis.

### Sample detection

Blood sample detection methods: FBG was determined by hexokinase assay (Beckman Coulter Automatic Biochemical Analyzer, AU5800, USA); FCP was determined by electrochemiluminescence assay (Roche Electrochemiluminescence Immunoassay Analyzer, Elecsys 2020, Switzerland); HbAlc was determined by high-performance liquid chromatography assay (Burroughs Glycosylated Hemoglobin Assay, BIO- D10, USA); TC was determined by enzymatic method; TG by GPO-POD method; LDL-C by direct method; HDL-C by analytical method; hs-CRP by immunoturbidimetric method; hs-CRP by enzymatic method. D10); TC was measured by enzymatic method; TG was measured by GPO-POD method; LDL-C was measured by direct method; HDL-C was measured by analytical method; hs-CRP was measured by immunoturbidimetric method. The homeostasis model assessment of insulin resistance (HOMA-IR) was calculated as HOMR-IR=1.5+FBG (mmol/L)×FCP (nmol/L)/2800.

Methods of gut microbiota analysis: DNA extraction, PCR amplification and sequence analysis: PCR amplification of the V4 region of the 16S rDNA gene was used to prepare sequencing libraries for high-throughput sequencing analysis (Shanghai BaoTeng Biomedical Technology Co., Ltd.). Raw sequences were obtained and screened for clustering of ASVs and species annotation. According to the abundance distribution of ASVs in different samples, we analyzed and compared the α-diversity and β-diversity of the gut microbiota between groups, and observed the community diversity and species richness, and the ecological structure of the gut microbiota. We also performed the analysis of differential gut microbiota between groups, PICRUSt prediction of differential functional metabolic pathways, and so on. Further statistical analyses were performed using the macrogenome mapping software package v2.1.3.

Metabolomics analysis methods: (1) Metabolite quantification: Tandem mass spectrometry coupled with high performance liquid chromatography (UPLC-MS/MS) system (ACQUITY UPLC-Xevo TQ-S, Waters CorpMilfordMA,USA) was used for the quantification of metabolites in this project. (2) Data processing and analysis: ①The raw data files generated by UPLC-MS/MS were processed using TMBQ software (v1.0, Metabo Profile, Shanghai, China) for peak integration, calibration and quantification of each metabolite. iMAP platform (v1.0, Metabo Profile, Shanghai, China) was used for statistical analysis, including PCA, OPLS-DA, univariate analysis and pathway analysis. ② Mass spectrometry-based quantitative metabolomics referred to the determination of the concentration of a substance in an unknown sample by comparing the sample with a set of standard samples (i.e., calibration curves) of known concentration. ③Multivariate statistical analysis: principal component analysis (PCA). ④ OPLS-DA (Orthogonal Partial Least Squares One Discriminant Analysis) was selected in this study to eliminate noise information not related to grouping and to screen for plausible metabolites that cause differences in grouping. The metabolites can be screened by VIP scoring through modeling analysis, and the higher the VIP score, the greater the contribution to the grouping. (3) Differential metabolite identification: ① Multi-dimensional statistics: Based on the results of the OPLS-DA model, the volcano plot (Volcanoplot) was used to screen reliable metabolic markers. The thresholds in the Volcanoplot were set as follows: VIP>1. (2) Unidimensional statistics: Unidimensional tests (TTest or Mann-Whitney U Test based on the normality and variance alignment of the data) were used to obtain the differential metabolites between the two groups. (4) Pathway analysis: using selected Pathway-associated metabolite sets (SMPDB) library, Predicted metabolite sets library, the differential metabolites were imported into the iMAP platform for pathway enrichment analysis; hsa library was utilized for pathway analysis.

### Statistical analysis

SPSS26.0 statistical data processing software was applied to analyze. Normality test and variance chi-square test were performed for all the measurement data, and the data with normal distribution of measurement data were expressed by mean earth standard deviation (SD), and skewed distribution by Mean (P25-P75). If the data belonged to normal distribution and variance chi-square the independent samples t-test was used between two groups, and ANOVA test was used between multiple groups. If the above conditions were not met, the two groups were compared using the Mann-WhitneyU nonparametric test, multi-group comparisons of the Kruskal-Wallis test; statistical description of the counting data using the rate or the composition of the ratio, and the comparison of groups using the chi-square test. Spearman analysis was used to analyze the correlation between gut microbiota, metabolites and biochemical indicators. Differences were considered statistically significant at *P*<0.05.

## Results

### Analysis of clinical data, glucose metabolism indexes, and levels of inflammatory factors in T2DM patients

Thirty patients with normal-weight T2DM and 30 patients with obese T2DM were included in this study. We found that T2DM patients did not have statistical differences in sex, age and height (*P*>0.05).The weight, BMI, SBP, waist circumference, FCP, HOMR-IR, and TG in the NNT group did not show significant abnormalities compared with those in the NCP group (*P*>0.05), but DBP, HbAlc, FBG, TC, and LDL-C were all higher than those in the NCP group (*P*<0.05), and HDL-C was significantly lower than that in the NCP group (*P*<0.05).The weight, BMI, DBP, waist circumference, HbAlc, FBG, FCP, HOMR-IR, TC, and LDL-C in the UNT group were higher than that in the NCP group (*P*<0.05), and HDL-C was significantly lower than that in the NCP group (*P*<0.001). Compared with the NNT group, weight, BMI, waist circumference, FCP, and HOMR-IR were elevated in the UNT group (*P*<0.05), and no significant abnormality of lipid metabolism indexes was observed between the two groups (*P*>0.05).In T2DM patients, HbA1c and FBG decreased in the NNT group after metformin treatment (*P*<0.05), while TC, TG, and LDL-C decreased compared to before, but the difference was not statistically significant (*P*>0.05).In the UNT group, BMI, HbA1c, FBG, HOMR-IR, TC, and LDL-C decreased after metformin treatment compared to before (*P*<0.05). Among the drug treatment groups, the concentrations of six inflammatory factors in the MET group were lower than those in the UNT group, with a statistical difference in the comparison of MCP-1, IL-6, and TNF-α (*P*<0.05) (**Table 1**).

The six indicators that responded to the systemic inflammatory state were higher in the UNT group and the NNT group than in the NCP group, in which the contrast of MCP-1, IL-6, TNF-α, and hs-CRP was significant (*P*<0.001); the contrast of resistin had a statistically significant difference (*P*<0.05), whereas the contrast of CXCL-1 was not statistically significant (*P*>0.05), and the concentration of the six inflammatory factors in the UNT group were all were higher than those in the NNT group, among which IL-6 was statistically different in comparison (*P*<0.05). Among the drug treatment groups, the concentrations of six inflammatory factors in the MET group were lower than those in the UNT group, with a statistical difference in the comparison of MCP-1, IL-6, and TNF-α (*P*<0.05), and the concentrations of six inflammatory factors in the NMT group were lower than those in the NNT group, but the difference did not have a statistical difference (*P*>0.05) (**Table 1**).

**Table 1:** Analysis of general data of T2DM patients.

	NCP (n=20)	NNT (n=30)	NMT (n=30)	UNT (n=30)	MET (n=30)	*p*
sex	8/12	13/17	13/17	14/16	14/16	0.264
age	46.20 ± 9.00	46.63 ± 6.90	46.63 ± 6.90	44.45 ± 5.95	44.45 ± 5.95	0.146
height	166.30 ± 7.55	166.98 ± 8.81	166.98 ± 8.81	169.08 ± 8.67	169.08 ± 8.67	0.732
weight	60.00(55.20 68.50)	62.11(55.45 67.65)^c**^	61.67(55 68)	85.55(79.25 90.08)^b**^	80.80(75.88 86.)	<0.001
BMI	22.60(61.00 23.22)	22.18(21.25 23.44)^c**^	22.18(21.55 22.86)	30.16(28.47 31.00)^b**^	28.15(26.55 29.48)^e*^	<0.001
waistline	81.09 ± 7.19	80.80 ± 4.03^c**^	81.04 ± 3.77	95.26 ± 4.78^b**^	93.42 ± 7.57	<0.001
SBP	116.0(111.0 125.5)	126.5(117 136)	127.57(122 135)	124.92(109 141)	127(110 136)	0.103
DBP	75(71 81)^a*^	83(74 89)	83(77.75 88)	82.96(72.75 96.75)^b*^	85(72 88.25)	0.093
HbA1c	5.10(5.05 5.50)^a**^	8.63(7.3 10.18)	7.00(6.13 7.88)^d*^	8.82(6.5 10.58)^b**^	7.50(6.4 8.98)^e*^	<0.001
FBG	4.97(4.73 5.38)^a**^	10.65(8.9 13.1)	7.10(7.26 11.33)^d**^	10.30(7.21 11.61)^b**^	7.61(6.64 9.49)^e*^	<0.001
FCP	0.62(0.45 0.77)	0.65(0.44 0.81)^c*^	0.63(0.45 0.77)	0.98(0.66 1.25)^b*^	0.89(0.71 1.34)	<0.001
HOM A-IR	2.61(2.27 2.98)	2.91(2.11 3.38)^c**^	2.97(2.65 4.26)	4.78(3.77 5.81)^b**^	4.01(3.08 5.45)	<0.001
TC	3.86(1.23 5.07)^a*^	5.11(4.21 5.84)	4.73(4.18 5.41)	4.94(4.20 5.72)^b*^	4.05(3.43 4.62)^e*^	0.003
TG	1.80(1.08 3.40)	2.14(0.96 2.77)	1.79(1.11 2.41)	2.49(1.44 2.88)	1.66(1.30 2.02)	0.415
HDL-C	1.36(1.11 2.02)^a**^	1.12(0.90 1.38)	1.14(0.91 1.32)	1.03(0.88 1.11)^b**^	1.00(0.84 1.17)	<0.001
LDL-C	2.27 ± 0.84^a**^	3.21 ± 0.93	2.98 ± 0.67	3.11 ± 0.84^b*^	2.42 ± 0.84^e*^	<0.001
MCP-1	102.78(87.5 112)^a**^	152.13(124.46 175.90)	149.15(128.06 165.03)	152.83(118.25 176.50)^b**^	128.00(88.00 140.50)^e*^	<0.001
IL-6	2.68(2.41 2.87)^a**^	3.46(3.04 3.45)^c**^	3.42(3.12 3.69)	4.28(3.42 4.70)^b**^	3.05(12.61 3.43)^e*^	<0.001
TNF-α	8.64(5.16 12.01)^a*^	10.69(9.03 11.73)	10.32(9.24 10.64)	13.26(9.34 14.75)^b*^	10.85(7.57 12.93)^e*^	0.001
CXCL-1	34.82(18.98 54.92)	37.13(29.48 41.08)	36.11(29.56 41.57)	39.62(23.48 50.88)	35.70(21.90 45.48)	0.431
resistin	10.64(5.59 12.21)^a*^	12.34(7.87 13.16)	11.69(4.91 17.37)	16.99(7.97 22.35)^b*^	14.41(8.05 20.15)	0.064
hs-CRP	0.71(0.35 0.97)^a**^	1.86(1.14 2.86)	1.65(1.22 2.49)	1.89(1.13 2.43)^b**^	1.55(0.87 1.90)	<0.001

NCP group, normal control persons; NNT group, normal-weight T2DM patients; NMT group, normal-weight metformin-alone treatment group; UNT group, obese T2DM patients; MET group: obese metformin-alone treatment group. p^a^: results of comparison between NNT group and NCP group; p^b^: results of comparison between UNT group and NCP group; p^c^: results of comparison between NNT group and UNT group; p^d^: results of comparison between NNT group and NMT group; p^e^: results of comparison between UNT group and MET group; * Represents P <0.05; * * represents P <0.001.

### Analysis of the gut microflora diversity of the study subjects

**Figure 1:**
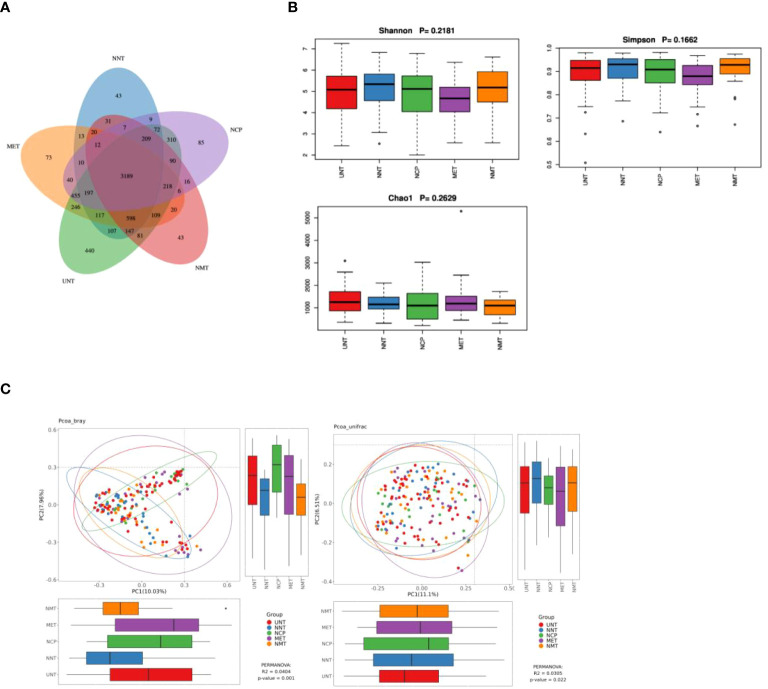
Analysis of the gut microflora diversity .**(A)** Venn diagram of ASVs for the five sample groups。**(B)** Comparison of α-diversity indices. Box plots depicting differences in fecal microbiome diversity among the five groups assessed using the Shannon, Simpson, and Chao1 indices, respectively, are shown. Each box plot represents the median, interquartile range, minimum, and maximum values. **(C)** *PCoA* analysis of five groups of gut microbiota *bray* analysis and *unweighted unifrac*. R-value, used to compare whether there is a difference between different groups; P-value, used to indicate whether there is a significant difference. R-value is between (-1, 1), R-value > 0 indicates that the difference between groups is greater than the difference within groups, R-value < 0 indicates that the difference between groups is less than the difference within groups, R is only a numerical indication of whether there is a difference between the groups and does not provide an indication of significance. The confidence level of the statistical analysis is expressed as P-value, with *P*< 0.05 indicating statistical significance.

Colony sequencing results: we collected a total of 140 samples from the five groups (**Figure 1A**), the overlapping ASV data in the Venn diagrams showed a total of 3,189 ASVs in the five groups, and the numbers of ASVs in the NCP, NNT, NMT, UNT and MET groups were 4,925, 4,781, 4,796, 6,585, 5323, respectively. The numbers of ASVs unique to each group were 85, 43, 43, 440, 73, respectively.

α-diversity analysis: the analysis showed that the differences in Shannon index, Simpson index, and Chao1 index among NCP, NNT, NMT, UNT and MET groups were not statistically significant (*P*>0.05) (**Figure 1B**).

β-diversity analysis: The *PCoA* plot of similarity level between the detected fecal microbial communities based on the *Bray-Curtis* distance showed that the contribution of each component was PC1 = 10.04%, PC2 = 7.96% (*P*=0.0404), and the *PCoA* plot of similarity level among the detected fecal microbial communities based on the *Unweighted Unifrac* distance made to detect the similarity level between fecal microbial communities *PCoA* plot showed that PC1 = 11.1% and PC2 = 6.51% (*P*=0.022). The results showed that the difference in β-diversity reflecting the similarity in the structure of the gut microbiota was statistically significant (*P*<0.05) among the five groups (**Figure 1C**).

**Figure 2:**
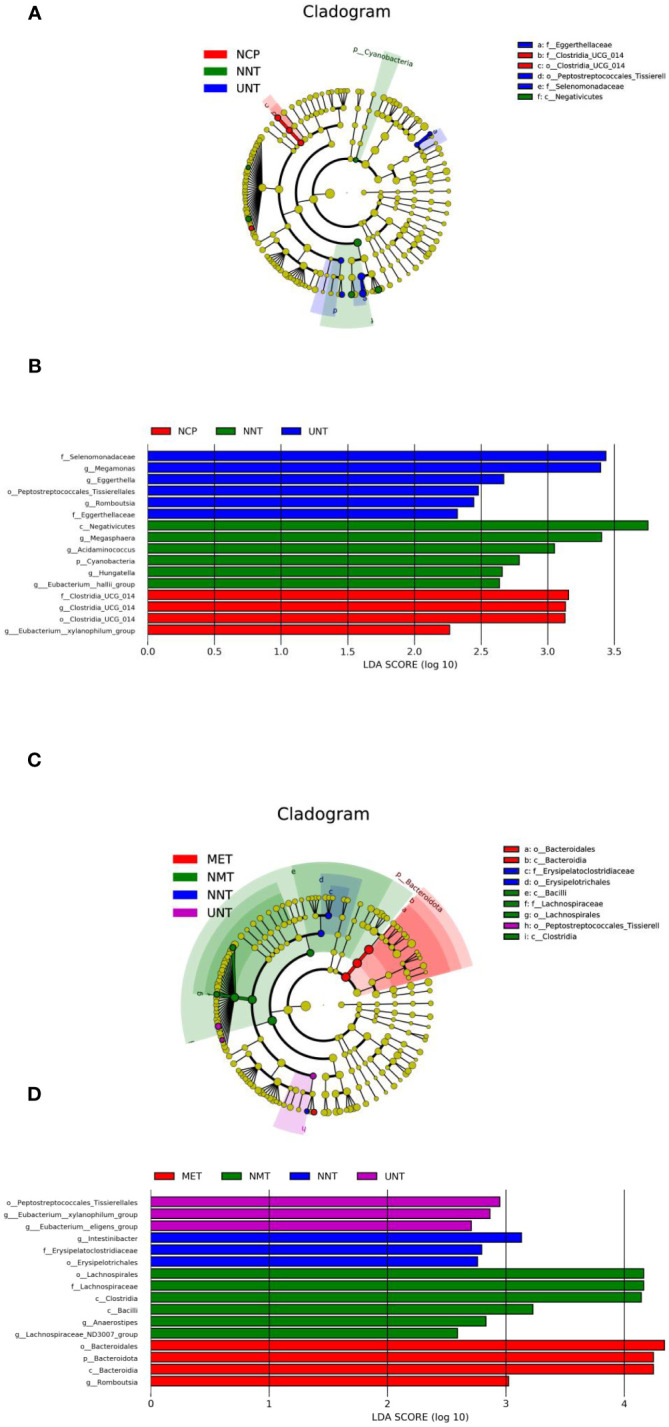
Bacterial features most likely explain differences among groups identified by LEfSe based on ASV level. The letter in the former of the name of bacteria indicates different taxa levels (‘‘g’’ indicates genus; ‘‘f’’ indicates family; ‘‘o’’ indicates order;’’c’’ indicates class; ‘‘p’’ indicates phylum). **(A)**Evolutionary branching diagram of LEfse analysis for three sets of samples of NCP, NNT and NMT. **(B)** LDA SCORE (log 10) plots for the three sets of samples of NCP, NNT and NMT. **(C)** Evolutionary branching diagram of LEfse analysis for four sets of samples of NNT, NMT, UNT and MET. **(D)** LDA SCORE (log 10) plots for the four groups of samples of NNT, NMT, UNT and MET.

Analysis of differential species between groups: according to the above analysis, the composition and structure of the gut microbiota of T2DM patients varied between groups. Therefore, in order to find reliable biomarkers, we used LEfSe analysis to assess intergroup differences. The results of the analysis were represented by taxonomic dendrograms and LDA histograms (LDA score >2), which showed the taxonomic hierarchical distribution of the species of the intestinal communities in each group and the species significantly enriched within each group and their level of importance, respectively. As shown in **Figures 2A, B**, the LEfSe results revealed 16 robust differential biomarkers among the three groups at taxonomic levels above genus level. The biomarkers with LDA scores > 2 in the NCP group were *f_Clostridia_UCG_014*, *g_Clostridia_UCG_014, o_Clostridia_ UCG_014*, *g_Eubacterium_xylanophilum_group*; similarly, the NNT group was significantly enriched for *c_Negativicutes*, *g_Megasphaera*, *g_Acidaminococcus*, *p_ Cyanobacteria*, *g_Hungatella*, *g_Eubacterium_hallii_group*; the UNT group screened for *f_Selenomonadaceae*, *g_Megamonas*, *g_Eggerthella*, *o_ Peptostreptococcales_Tissierellales*, *g_Romboutsia*, *f_Eggerthellaceae*.

As shown in **Figures 2C, D**, *o:Erysipelotrichales, f:Erysipelatoclostridiaceae, g:Intestinibacter were* significantly enriched in the NNT group, *g:Lachnospiraceae_ND3007_ group, g:Anaerostipes, c:Bacilli, c:Clostridia, f:Lachnospiraceae, o:Lachnospirales were enriched in the* NMT group, *g:Eubacterium:eligens_group, g: Eubacterium:xylanophilum_group, o:Peptostreptococcales_Tissierellales were* significantly enriched in the UNT group, *g:Romboutsia, c:Bacteroidia, p:Bacteroidota, o: Bacteroidales were enriched in the* MET group.

**Figure 3:**
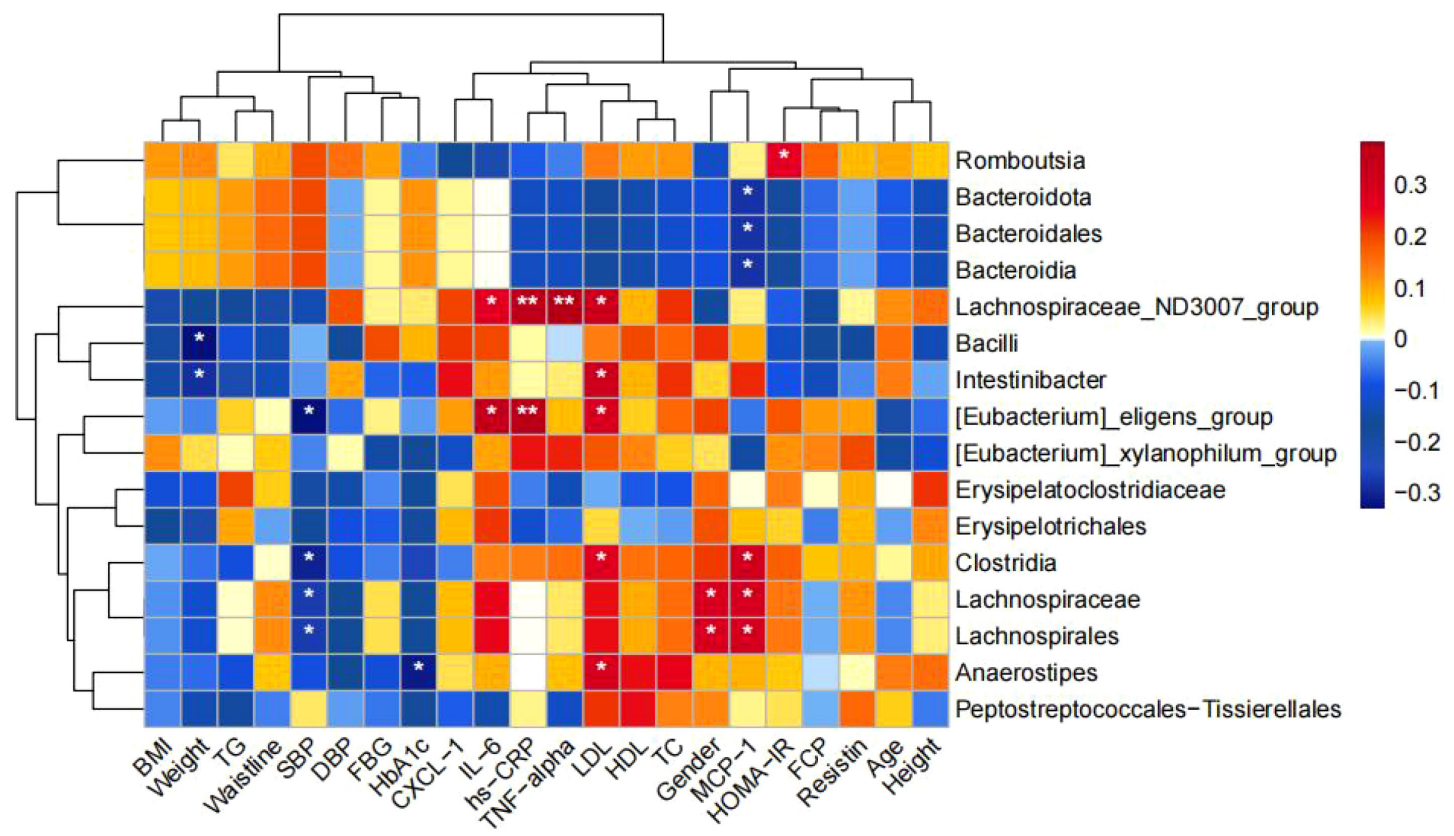
Correlation between the four groups comparing differential bacteria and clinical indicators. (*p<0.05, **p<0.01).

Correlation analysis between Differential Bacteria and Clinical Indicators: **Figure 3** *g_Intestinibacter* enriched in NNT group was negatively correlated with waist circumference and positively correlated with LDL; *g_Anaerostipes* enriched in NMT group was negatively correlated with HbA1c and positively correlated with LDL; *g_Eubacterium_eligens_group* significantly enriched in the UNT group was positively correlated with LDL, IL-6, and hs-CRP; *Romboutsia* significantly enriched in the MET group was positively correlated with HOMA-IR; and *Bacteroidota* was negatively correlated with MCP-1.

**Figure 4:**
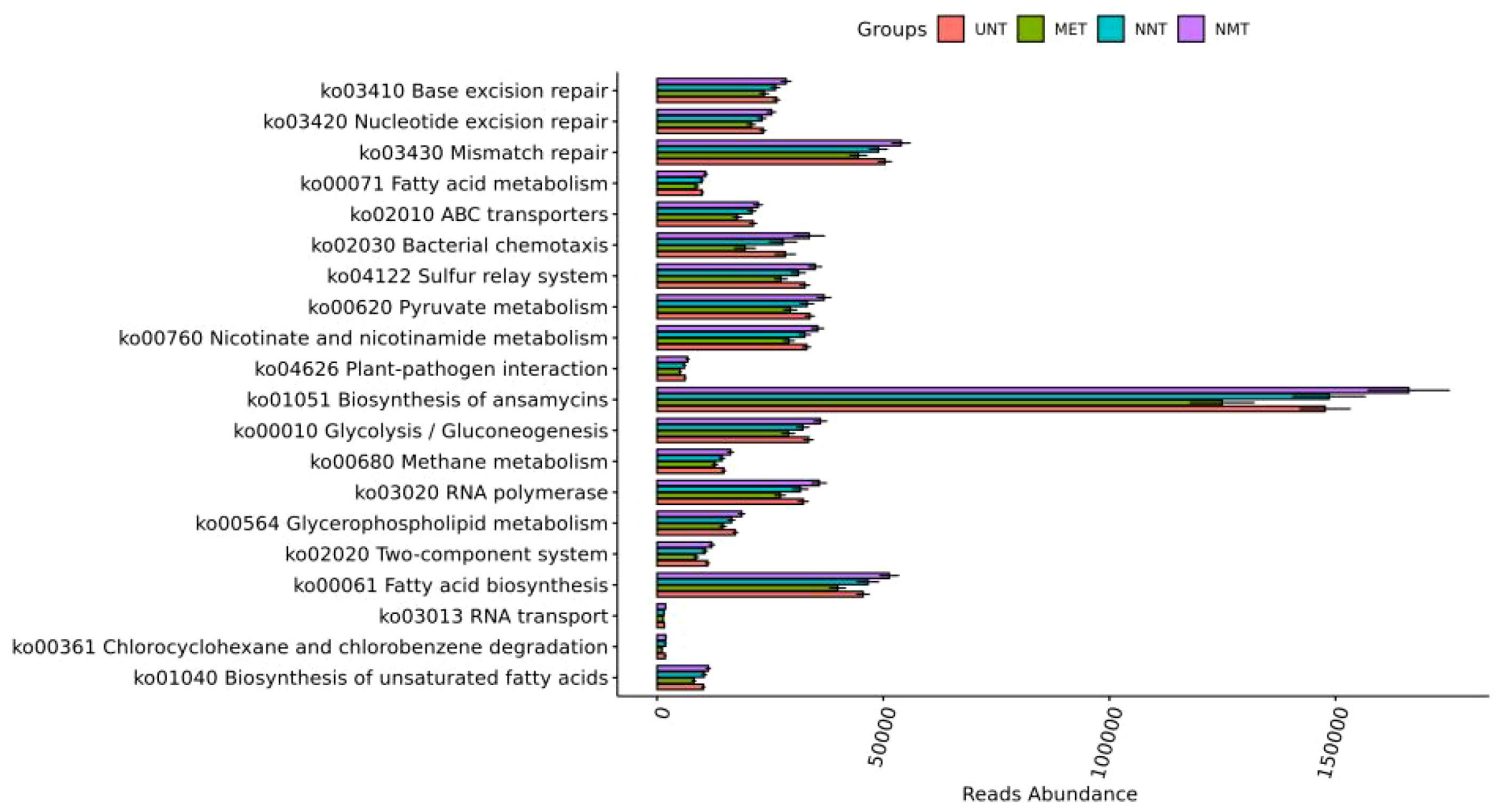
Comparison of KEGG pathway analysis results.

Functional prediction: the results of KEGG pathway analysis were shown in **Figure 4**, and the most different pathways among the four groups were: fatty acid biosynthesis, chlorocyclohexane and chlorobenzene degradation, glycerophospholipid metabolism, and unsaturated fatty acid biosynthesis, and the differences were statistically significant (*P*<0.001).The most different pathways between the MET and NMT groups were: unsaturated fatty acid biosynthesis, chlorocyclohexane and chlorobenzene degradation, binary signaling system, bacterial chemotaxis, and glycerophospholipid metabolism.

### Analysis of metabolomics results

**Figure 5:**
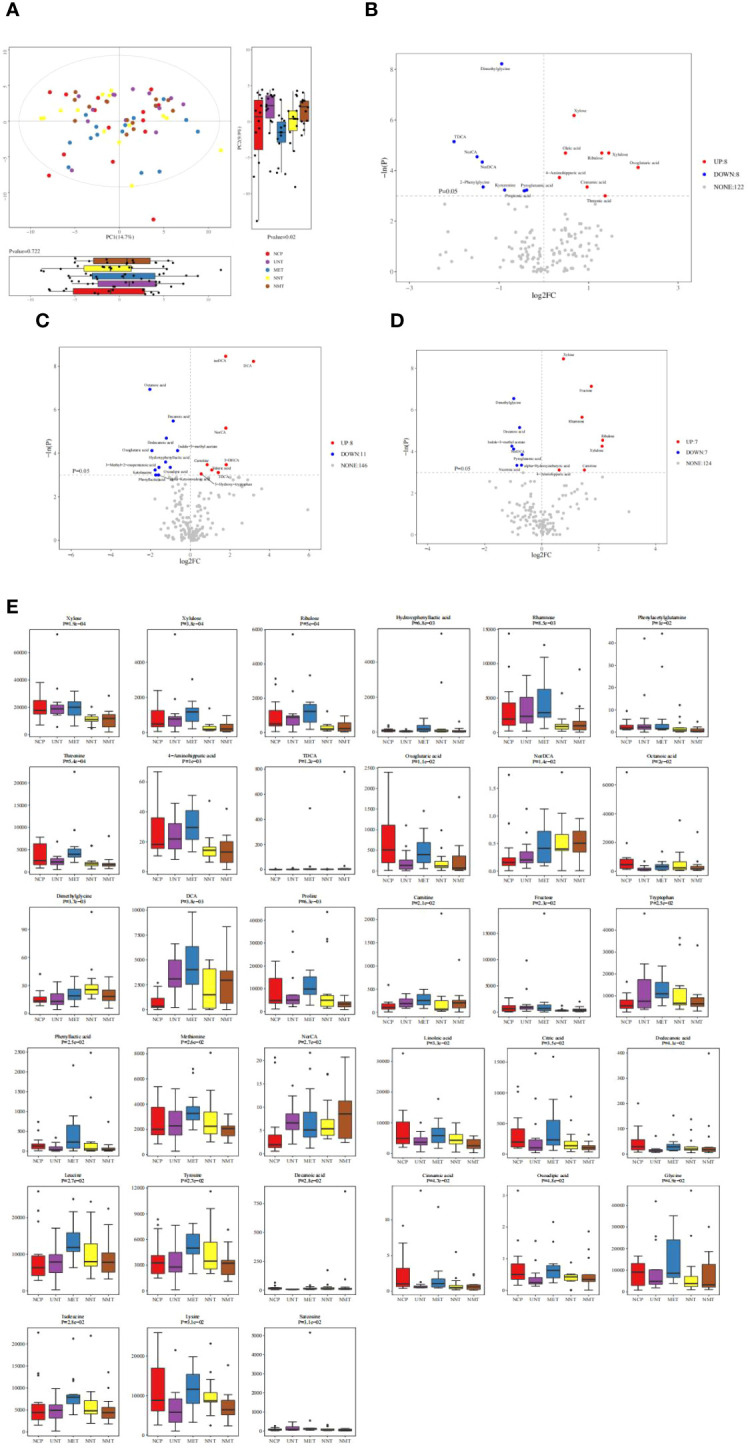
Analysis of metabolomics results. **(A)** Principal component analysis graph. The figure shows a plot of the 2D principal component scores of the analyzed samples and a box plot of the corresponding principal component scores. The box plots provide a more intuitive view of the differences in the first and second principal component scores for different groups of samples. Each point in the graph represents a sample, and different colors indicate different groups. The principal components shown in the figure are the combinations of principal components that have the largest distance from each other among all the subgroups. The percentage in parentheses after the principal component represents the overall rate at which that component explains the data. **(B–D)**The vertical dashed line indicates the dotted line corresponding to the FC threshold taken logarithmically and log2FC as the horizontal coordinate; the horizontal dashed line indicates the dotted line corresponding to *P*=0.05 and the corresponding -logeP value as the vertical coordinate. Meanwhile, the points that meet the requirements above the horizontal dashed line and on both sides of the vertical dashed line will be highlighted, **(B)** where the red highlights on the right side indicate the metabolites whose concentration increased, i.e., up-regulated, in the NCP group of the observation group compared to the NNT group of the control group and the blue highlights on the left side indicate the metabolites whose concentration decreased, i.e., down-regulated, in the NCP group of the observation group compared to the NNT group of the control group; **(C)** where the red highlights on the right side indicate the metabolites that were up-regulated by increasing concentrations in the UNT group compared to the control NCP group and the metabolites that were down-regulated by decreasing concentrations in the UNT group compared to the control NCP group. **(D)**The blue highlights on the left side indicate metabolites that were down-regulated by decreasing concentrations in the UNT group compared to the control NNT group; the gray dots indicate metabolites that did not meet the requirements of the set threshold. **(E)** Comparison of differential metabolites among the five groups.

Principal component analysis: in this work, targeted metabolomics was applied to explore the intestinal metabolic profile of T2DM patients. As shown in **Figure 5A**, after principal component analysis, NCP, NNT, NMT, UNT and MET groups showed different distribution trends, with no significant difference in the first principal component (*P*>0.05) and significant difference in the second principal component (*P*=0.02).

Differential metabolite identification: volcano plots showed the relative changes in differential metabolites in the two groups. As shown in **Figures 5B–D**, 16 differential metabolites were obtained in gut metabolites of normal-weight T2DM patients and 19 differential metabolites were obtained in gut metabolite species of obese T2DM patients compared to healthy controls. 14 differential metabolites were obtained in gut metabolites of obese T2DM patients and normal-weight T2DM patients. The metabolites that were elevated in the normal-weight T2DM group compared to both the obese T2DM group and healthy controls were: dimethylglycine and nordeoxycholic acid; and the metabolites that were decreased in the normal-weight T2DM group compared to both the obese T2DM group and healthy controls were: xylose, ribulose, xylulose, and 4-aminoimauric acid. The box plots in **Figure 5E** showed the differential metabolites in the gut metabolites of T2DM patients before and after treatment with metformin for four comparisons of groups: the metabolites that were increased in the NNT that showed a more pronounced decrease in the NMT group were: dimethylglycine. the metabolites that were decreased in the NNT that showed a more pronounced increase in the NMT group were: xylose, ribulose, and xylulose.

**Figure 6:**
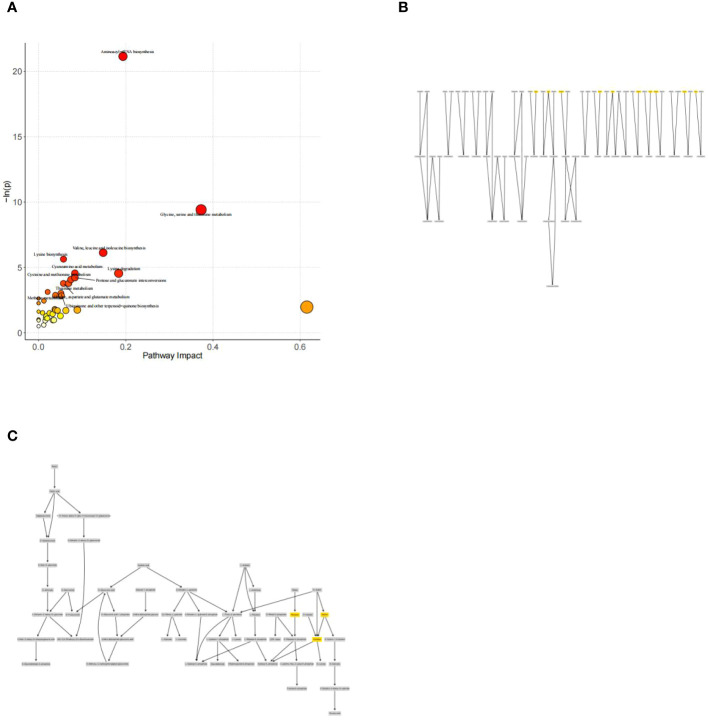
**(A)** Metabolic pathway and influence analysis. Each circle corresponds to a metabolic pathway, the horizontal coordinate indicates the degree of pathway impact, the size of the circle is related to the pathway impact of the pathway, the larger the Impact value the larger the circle, the vertical coordinate indicates the negative logarithm of the P-value obtained from the enrichment analysis of the pathway, and the change of the yellow-red color of the point is positively related to the negative logarithm of the P-value of the pathway change. Correlation. Pathways with *P*<0.05 are labeled with their names in the figure, and pathways that do not meet the above conditions are not labeled with their names in the figure. **(B)** Network diagram for pathway analysis of aminoacyl-tRNAs biosynthesis. **(C)** Network diagram for pathway analysis of Pentose and glucuronate interconversions.

Analysis of metabolic pathways associated with diabetes mellitus: The major metabolic pathway abnormalities found were Aminoacyl-tRNA biosynthesis (*P*<0.05); Glycine, serine and threonine metabolism (*P*<0.05); Valine, leucine and isoleucine biosynthesis (*P*<0.05); Lysine degradation(*P* < 0.05); and Pentose and glucuronate interconversions (*P*<0.05). ((**Figure 6A**). Amino acids involved in aminoacyl-tRNAs biosynthesis mainly include amino acids such as glycine, serine, methionine, lysine, alanine, isoleucine, leucine, threonine, and tyrosine.(**Figure 6B**). And xylose, ribulose, and xylulose were mainly involved in Pentose and glucuronate interconversions.(**Figure 6C**).

**Figure 7:**
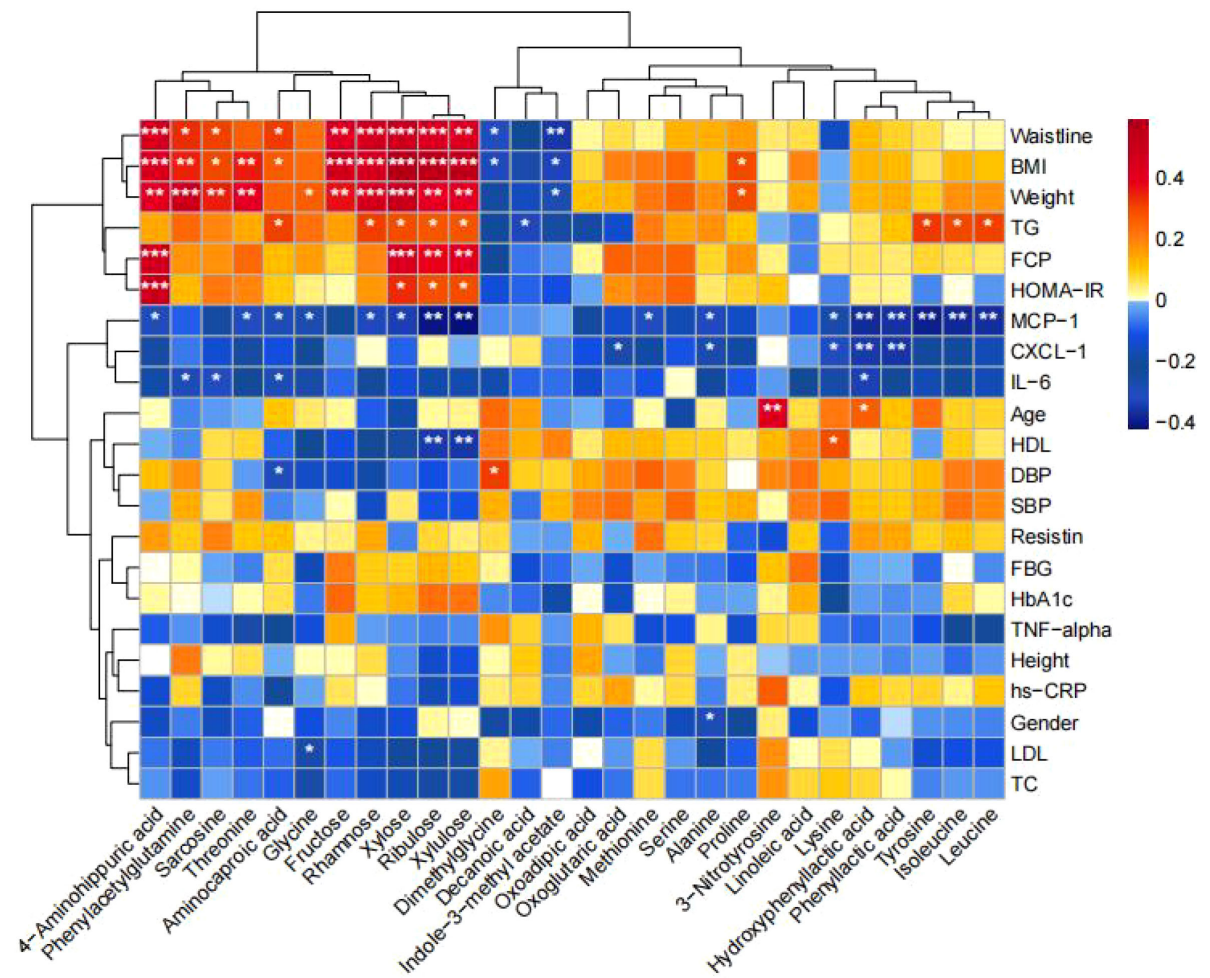
Correlation between differential metabolites and clinical indicators. Heatmaps show the correlation between differential metabolites and clinical indicators. Red color represents positive correlation and blue color represents negative correlation. The darker the color, the stronger the correlation. (*p<0.05, **p<0.01, ***p<0.001).

Correlation between differential metabolites and clinical indicators: the fecal differential metabolites obtained from the comparison of the four groups were subjected to *spearman* correlation analysis with clinical indicators, as shown in **Figure 7**, and it was found that xylose was positively correlated with BMI, waist circumference, body weight, FCP, HOMA-IR, TG, and negatively correlated with MCP-1; and that ribulose and xylulose were correlated with BMI, waist circumference, body weight, FCP, HOMA-IR, TG were positively correlated and negatively correlated with MCP-1 and HDL-C.

### Association between altered gut microbiota and metabolites

**Figure 8:**
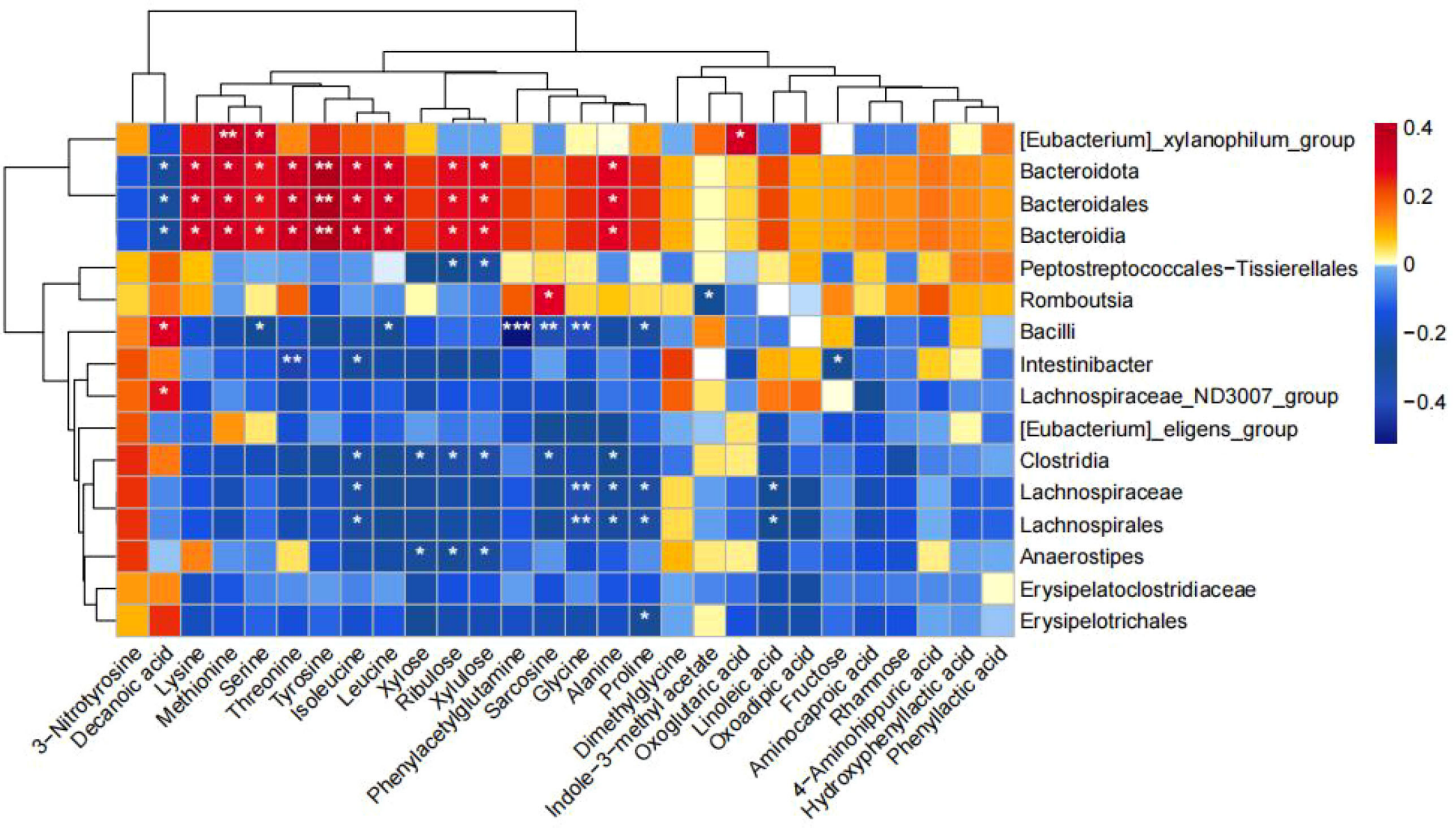
Correlation between changes in fecal metabolites and changes in gut bacteria abundance. Heatmap showing the correlation between changes in fecal metabolite concentrations and changes in the relative abundance of enteric bacteria. Red color represents positive correlation and blue color represents negative correlation. Darker colors indicate stronger correlations. (*p<0.05, **p<0.01, ***p<0.001).

As shown in **Figure 8**, we found that *Intestinibacter* enriched in the NNT group was negatively correlated with Fructose, isoleucine, and Threonine; *g_Anaerostipes* enriched in the NMT group was negatively correlated with Xylose, Ribulose, and Xylulose; and *c_Clostridia* was negatively correlated with sarcosine, Xylose, Ribulose, Xylulose, and isoleucine.

## Discussion

T2DM is a common chronic metabolic disease characterized by hyperglycemia due to relative insulin deficiency (12).T2DM accounts for more than 95% of all diabetes mellitus (13), and is an important cause of diabetic complications and high mortality in diabetic patients (14, 15). The risk factors and glucose metabolism characteristics of obesity and diabetes have been widely reported, but the current reports on the characteristics of normal-weight T2DM are still limited. Currently, the physiological mechanisms that promote the progression of T2DM are not fully understood. With the rapid development of sequencing and spectroscopic technologies, the role of gut microbiota and its metabolites in disease progression, it is believed that the gut microbiota and its metabolites are a possible factor influencing the pathogenesis of metabolic diseases. Therefore, therapeutic and preventive strategies by regulating the gut microbiota and metabolites in type 2 diabetes mellitus patients are well worth researching and exploring. Current guidelines at home and abroad recommend metformin as a first-line therapeutic agent for T2DM (16), which has the ability to regulate glycolipid disorders, antioxidant and anti-inflammatory effects, modulate glycolipid metabolism and improve insulin resistance by reducing oxidation and inflammation. The presence of dysbiosis in patients with T2DM at first diagnosis is mainly characterized by an increase in the overall activity of the gut microbiota and, at the same time, significant changes in the proportions of some specific genera (17). Therefore, exploring and discovering the effects of metformin on glucose metabolism, lipid metabolism and gut microbiota and their metabolites is important for the prevention and treatment of diabetes. Previous animal experiments and clinical trials had shown that metformin can affect the gut microbiota and its metabolites (6, 18, 19), but studies had mostly focused on obese patients. In contrast, we compared the effects of metformin normal-weight T2DM gut microbiota. In this study, we observed the characteristics of gut microbiota and changes in metabolite fractions in normal-weight T2DM patients by 16S rDNA gene sequencing technology and high performance liquid chromatography-mass spectrometry-targeted metabolomics, and we explored the effects of gut microbiota and their metabolites on glucose and lipid metabolism of the organism after metformin treatment, which can provide a basis for further revealing the relationship between diabetes mellitus and gut microbiota and for guiding the normal-weight T2DM patients with provide the basis for precise treatment program.

Our study found that HbA1c, FBG, FCP, HOMA-IR, TC, LDL-C, were higher and HDL-C was lower in patients with T2DM than in the healthy population, and that weight, BMI, and waist circumference were significantly lower in the healthy population and in the normal-weight T2DM patients than in the obese T2DM patients. Meanwhile, we found that the treatment of T2DM patients with metformin could improve the glycolipid metabolism, but the recovery of glycolipid metabolism was more obvious in obese T2DM patients, which may be due to the fact that no weight loss is required for normal-weight T2DM patients and that there exists stricter dietary and exercise interventions for obese T2DM patients. We also compared six inflammatory indicators: MCP-1, IL-6, TNF-α, CXCL-1, Resistin, and hs-CRP were higher in T2DM patients than in the healthy population, but the concentrations of all six inflammatory factors were lower in normal-weight T2DM patients than in obese T2DM patients. This may be related to the fact that both obesity and diabetes are a low-grade inflammatory state, and the degree of inflammation is increased when the two are present in combination. This is consistent with the results reported in the current study (20).Meanwhile, we also compared the six inflammatory indicators in T2DM patients after metformin treatment and found that all of them showed a decreasing trend, which suggests that the reduction of the inflammatory state in the body of T2DM patients after drug treatment may be related to the recovery of their blood glucose level and pancreatic islet function.

We sequenced samples for 16S rDNA gene sequencing. The diversity of the gut microbiota of normal-weight T2DM patients was verified by α and β diversity analysis. The data showed that species diversity and community richness were higher in normal-weight T2DM patients than in the healthy population, and the overall structure was altered, but not differentially. To further assess the precise changes in the gut microbiota, we performed LefSe to analyze the differential bacteria between groups and found that normal-weight T2DM patients were enriched in *c_Negativicutes*, *g_Megasphaera*, and *g_Acidaminococcus*, and obese T2DM patients were enriched in the gut microbiota in *g_Megamonas*, *g_ Eggerthella*, *g_Romboutsia*, and *f_Eggerthellaceae* were higher than those of the healthy population, while *Clostridia* were lower than those of the healthy population. *Acidaminococcus* belongs to the order *Negativicutes* and *Firmicutes*, with glutamate as the main energy source. *Acidaminococcus* has been positively associated with T2DM risk factors, such as fasting blood glucose and HbA1c (21), and has shown a significant positive correlation with cholesterol and triglyceride levels, suggesting that it may be involved in fatty acid metabolism. *Megasphaera*, also belonging to Firmicutes, ferment mainly fructose and lactic acid, and a previous macro-genomic study found higher abundance of *Megasphaera* and lower abundance of *Clostridia* in patients with T2DM and pre-diabetes compared to metabolically healthy controls (22), which is consistent with our study, whereas *Clostridia* is recognized as a probiotic associated with tryptophan metabolism in mice with colitis, and may mediate intestinal barrier disruption, colon inflammation and amelioration of pathological phenotypes (23). In conclusion, our study corroborated the previous characterization of the gut microbiota in the T2DM population, but we also found that there are differences in the changes of gut microbiota *in vivo* for the first diagnosis of T2DM, suggesting that attention needs to be paid to bacterial duality and dynamic changes in future studies. We tested the intestinal microecological balance of the gut microbiota after metformin intervention and compared it with that at baseline, and the data showed that, although it was more similar in terms of community diversity and species richness, the community diversity and species richness were restored after metformin intervention compared with the previous one, which was closer to that of healthy population. This shows that metformin can improve intestinal dysbiosis, which indicates the rationality of clinical glucose lowering for T2DM from the perspective of gut microbiota, which is also a strong proof of the guidelines’ heavy recommendation of the first-line drug. Meanwhile, in the analysis of differential bacteria between groups, we found that normal-weight T2DM patients were enriched in the metformin-treated group with one kind of bacterium*, g_Anaerostipes*, which was negatively correlated with HbA1c and positively correlated with LDL-C as suggested by the spearman’s correlation with the clinical indexes. The genus *Anaerostipes* is one of the most enriched taxa in the healthy microbiota and one of the butyric acid producing and is one of the most efficient lactic acid consuming bacteria in the human gut microbiota. The abundance of *Anaerostipes* has been reported to be significantly lower in African and European T2DM patients (24, 25). More than half of *Anaerostipes* are able to use inositol as the sole source of carbon and energy and can convert dietary inositol to propionate (26), which has been suggested to have an indirect effect on diabetes by lowering adipogenesis and serum cholesterol levels, thereby decreasing the risk of diabetes development (27). In contrast, in obese T2DM patients, *Romboutsia*, enriched after metformin treatment, was positively correlated with HOMA-IR; *Bacteroidota* was negatively correlated with MCP-1. This suggests that metformin may act through different gut microbiota in normal-weight versus obese T2DM. In summary, we speculated that *Anaerostipes* was a beneficial bacterial genus that plays a role in glycemic dyslipidemia and anti-inflammation, and we can use this as a target for further animal experiments for validation in the future.

Our correlation analysis of gut microbiota with clinical biochemical indices revealed that *Megasphaera* enriched in normal-weight T2DM was positively correlated with FBG, HbA1c, hs-CRP, and MCP-1, *Negativicutes* was positively correlated with FBG and HbA1c, and *Acidaminococcus* was negatively correlated with HDL-C. *Megamonas* enriched in obese T2DM patients was positively correlated with BMI, waist circumference, and HOMA-IR; *Eggerthella* was positively correlated with TNF-α, hs-CRP, resistin, IL-6, and LDL-C. *Clostridia_UCG_014* enriched in healthy controls was negatively correlated with FBG, HbA1c, IL-6, and MCP-1. Consistent with previous studies, this further suggests that gut microbiota may play an important role in the disorders of glycolipid metabolism and inflammatory effects in T2DM patients.

We also observed trends in metabolic pathways: a decrease in aminoacyl tRNA biosynthesis, metabolism of glycine, serine, and threonine, biosynthesis of valine, leucine, and isoleucine, and lysine degradation pathways. Aminoacyl tRNA biosynthesis is involved in the synthesis of amino acids as well as in a variety of metabolic processes such as protein synthesis, hormone synthesis, and glycolipid metabolism (28).Roas et al. found a significant enrichment of metabolites associated with aminoacyl-tRNA biosynthesis after the use of metformin (29), and in our study we found that the metabolism of a wide variety of amino acids centered on aminoacyl- tRNA biosynthesis mainly including amino acids such as glycine, serine, threonine, methionine, lysine, alanine, isoleucine, leucine, and tyrosine, and that a decrease in the metabolic pathways of glycine, serine, and threonine indicated an increase in the levels of glycine, serine, and threonine, which was in agreement with the previous study (30), in which glycine, serine, and threonine were associated with an improvement in insulin sensitivity (31). Previous studies have shown that changes in plasma glycine may be one of the biomarkers of T2DM (32), and Chen et al. found in their study that insulin secretion was higher in diabetic rats taking glycine compared to diabetic rats not taking glycine (33). It was also found that glycine improved the microstructure of pancreatic β-cells and increased the number of mature insulin-secreting granules. Serine is a non-essential amino acid that plays a role in the metabolism of fats and fatty acids and the growth of muscles, and can lower cholesterol levels, helping to prevent diseases such as hypertension and atherosclerosis. Threonine is an essential amino acid that promotes protein synthesis, maintains cell function, promotes liver metabolism, and improves liver detoxification to a certain extent. While metformin can inhibit gluconeogenesis, its mechanism may rely on the AMPK-dependent pathway (34), it was found that metformin can promote the phosphorylation of serine/threonine kinase 11, which phosphorylates T172 on the α1 subunit of AMPK and activates AMPK and inhibits the adverse effects of hepatic protein kinase B1 on metformin. AMPK Activation triggers cAMP catabolism to reduce glucagon stimulated cAMP and PKA signaling. cAMP and PKA signaling is diminished, glycogen synthesis is enhanced, and gluconeogenesis is inhibited. Valine, leucine, and isoleucine are branched-chain amino acids, and Huda et al. found that insulin-resistant patients exhibit abundant biosynthesis of branched-chain amino acids and were found to lack the genes encoding bacterial inward transporter proteins for these specific amino acids. Phosphorylation of insulin receptor substrate-1 on serine residues by stimulating rapamycin and its downstream effector mTOR/S6 kinase interferes with insulin signaling (35). Therefore, it can be assumed that amino acids play an important role in glucose homeostasis, and supplementation of amino acids, a metabolite, can improve glucose tolerance, and, metformin may regulate the disorders of glucose-lipid metabolism in the T2DM population through amino acid-related pathways, but the mechanisms of these metabolic pathways associated with the hypoglycemic effects of metformin remain to be further investigated. Fecal metabolomics reveals that metformin treatment reverses metabolic abnormalities in normal-weight or obese T2DM patients. Metformin treatment upregulated metabolites that were decreased in normal-weight T2DM patients: xylose, ribulose, and xylulose, and their correlation with clinical indicators was found to be positively correlated with BMI, waist circumference, and body weight in the correlation study. And the metabolites up-regulated in obese T2DM patients were: octanoic acid, decanoic acid, dodecanoic acid, and decanoic acid was negatively correlated with TG. In order to further clarify the association between gut microbiota and these metabolites, we continued the *spearman* analysis of gut microbiota with metabolites, and we found that *Anaerostipes* were negatively correlated with xylose, ribulose, and xylulose; and *Clostridia* were negatively correlated with sarcosine, xylose, ribulose, xylulose, and isoleucine. This suggests that metformin may exert its effects in normal-weight versus obese T2DM patients possibly through different gut microbiota and metabolites.

Gut microorganisms interact with the host by producing different metabolites. Therefore, we used UPLC-MS/MS to quantify targeted Q200 macro-metabolomics in fecal samples. The metabolites that were significantly reduced in normal-weight T2DM patients compared to obese T2DM and healthy controls consisted of pentose, glucuronic acid metabolism, mainly xylose, ribulose, and xylulose. In contrast, the metabolites that were significantly reduced in the intestines of obese T2DM patients compared to normal-weight T2DM and healthy controls were mainly composed of lipid metabolism, such as octanoic acid, dodecanoic acid, and decanoic acid. On the contrary, the metabolites that were significantly increased in obese T2DM patients compared to normal-weight T2DM and healthy controls consisted mainly of bile acid metabolism, mainly DCA, NorCA. xylose, ribulose and xylulose all belong to pentose, while pentose and glucuronic acid are two common glycoconjugates, which are important for research in biochemistry, medicine, and other fields. Xylose is a component of xylan with anti-bacterial and anti-mold properties; it helps to inhibit the growth and reproduction of harmful microbiota in the gut and enhances the growth of beneficial microbiota in the gut, such as *Bifidobacterium*, which has significant health benefits, including improved intestinal permeability, which leads to lower circulating levels of endotoxins and reduced systemic inflammation. This has been linked to improved glucose tolerance and glucose-induced insulin secretion in the host and reduced inflammation (36). Most importantly, xylose also has hypoglycemic properties that help promote insulin secretion in the body, reduce blood glucose levels and control the onset or progression of metabolic diseases such as diabetes. Ribulose is a monosaccharide with a pentose structure corresponding to ribose, which can appear in the reductive pentose phosphate cycle in photosynthesis. Su Tao et al. found that T2DM not only had significantly higher urinary glucose concentrations than normal controls, but also significantly higher urinary pentose concentrations, suggesting that not only glucose metabolism but also pentose metabolism was abnormal in T2DM (37). It had also been found that intravenous pentose could significantly increase serum insulin level and maintain it for a long period of time, and its effect of lowering blood glucose was only significant after 5 minutes of intravenous pentose, and the effect of lowering blood glucose gradually disappeared with the prolongation of time (38). In the present study, we found that the major metabolic pathways of normal-weight T2DM distinguishing healthy populations by pathway analysis of differential metabolites in the hsa library were: pentose, glucuronide interconversion, fatty acid biosynthesis, starch and sucrose metabolism. We also identified pentose as part of the potential biomarkers associated with the pentose phosphate pathway. Thus, pentose promotes both energy metabolism and improves energy metabolism in ischemic and hypoxic cells, as well as promotes insulin production and lowers blood glucose. Octanoic acid, dodecanoic acid, and decanoic acid belong to medium-chain fatty acids, and related studies have also found that medium-chain fatty acids can reduce T2DM-induced hyperlipidemia, insulin resistance, oxidative stress, and inflammatory response, repair liver function damage, and promote glycogen synthesis. It also activates PI3K/AKT/GLUT-2 signaling pathway, promotes glucose metabolism gene expression and maintains glucose homeostasis. And the correlation study with clinical indicators found that Dimethylglycine was positively correlated with age, DBP, LDL-C, TC, hs-CRP, and NorDCA was positively correlated with FBG, HbA1c, HOMA-IR, DBP, LDL-C, TC, IL-6, resistin, hs-CRP; xylose was positively correlated with FBG, HbA1c, HOMA-IR, DBP, LDL-C, TC, IL-6, resistin, hs-CRP. Xylose was positively correlated with waist circumference and negatively correlated with age. Decanoic acid was negatively correlated with waist circumference, BMI, TG, and positively correlated with HDL-C; Octanoic acid was negatively correlated with waist circumference, BMI, TG, FBG, HbA1c, IL-6, and positively correlated with HDL-C; and Dodecanoic acid was negatively correlated with waist circumference, BMI, TG, FBG, HbA1c, and positively correlated with HDL-C. This all suggests that changes in intestinal metabolites may all play an important role in the development of metabolic diseases such as T2DM, and that different metabolites may play a role in obesity and normal-weight T2DM.

In conclusion, we used 16S rDNA sequencing technology and Q200-targeted macro-metabolomics approach to understand changes in gut microbiota and its metabolite fractions and functional metabolic pathways in patients with T2DM compared to healthy controls. It was determined that there were significant overlaps and differences in the composition and functional characteristics of the gut microbiota in the normal-weight T2DM group, the obese T2DM group and the healthy control group. On this basis, we found that *c_Negativicutes, g_Megasphaera*, xylose, ribulose and xylulose play important roles in the development of normal-weight T2DM, and in obese T2DM, *g_Megamonas, g_Eggerthella, g_Romboutsia*, decanoic acid, octanoic acid, and dodecanoic acid may be specific microbiota and metabolites that play important roles in the pathogenesis of obese T2DM. Similarly, we found that *Anaerostipes*/xylose/ribulose/xylulose may play an important role in the treatment of normal-weight T2DM with metformin by improving glycemic lipids and reducing inflammation. However, the related microbiota and metabolites have been less studied in the population, especially in normal-weight type 2 diabetes mellitus, which deserves to be verified in future animal experiments as well as related population intervention trials.

This study has some limitations. First, the small sample size of this study cannot represent the relevant changes of microbiota and metabolites in T2DM patients at different BMI, so further large-scale longitudinal, interventional and multicenter studies are needed in the future. Second, in recent years, several papers have reported the effects of diet and exercise on gut microbiota and metabolites, and although we provided health education to our patients, we were not able to completely avoid this influencing factor due to the fact that no weight loss is required for normal-weight T2DM patients and that patients have limitations such as self-control and work and other related factors, which may have an impact on our results. Finally, we selected T2DM patients with a short course of the disease, and our results are not applicable to T2DM patients with a longer course of the disease.

The strength of this study is that we successfully applied 16S rDNA sequencing technology and Q200 quantitative macrometabolomics to reveal the characteristics of intestinal microecological dysregulation and metabolic disorders in patients with normal-weight T2DM, and we also found that metformin could intervene in normal-weight T2DM by reversing the abnormalities of pentose, amino acids, and other related metabolisms and by modulating interactions between metabolites and gut microbiota. In conclusion, the We selected normal-weight T2DM patients with short disease duration and included obese T2DM patients to analyze the role of weight in the gut microbiota and its metabolites in T2DM patients, to further understand the gut microbiome-metabolome interactions in normal-weight T2DM patients and to systematically elucidate the pathogenesis of T2DM from the perspective of the host microbial metabolic axis and metformin’s possible Therapeutic mechanisms.

## Conclusion

In summary, we analyzed and compared the microbiota characteristics of normal-weight T2DM, and we hypothesized that the gut microbiota affect the host through metabolites, which provides further understanding of microbiome-metabolome interactions in T2DM, and may be useful for the future diagnosis and treatment of T2DM, adding to this field. In addition, we combined 16S rDNA sequencing technology and Q200 quantitative macrometabolomics and found that metformin treatment normalized the diversity and abundance of the gut microbiota and its metabolites in normal-weight type 2 diabetic patients. It may be related to *Anaerostipes-*xylose/ribose/xylulose. Correlation analysis between gut bacteria and metabolites not only highlights their relationship, but also helps to explain the pathogenesis of normal-weight T2DM and potential mechanisms of drug therapy.

## Data Availability

The datasets presented in this study can be found in online repositories. The names of the repository/repositories and accession number(s) can be found below: https://www.ncbi.nlm.nih.gov/bioproject/PRJNA1133738.
